# Fluorescent bioreporters for assessing nitrogenase expression and rhizobial nodule occupancy in *Lotus japonicus* and *Pisum sativum*

**DOI:** 10.1128/mra.00103-26

**Published:** 2026-03-23

**Authors:** Gayathri Senanayake, Amanda B. Pease, Chandan K. Gautam, Birgit M. Prüß, Barney A. Geddes

**Affiliations:** 1 Department of Plant Pathology, Microbiology and Biotechnology, North Dakota State University 3323, Fargo, North Dakota, USA; Rochester Institute of Technology, Rochester, New York, USA

**Keywords:** biosensor, symbiosis, nitrogen fixation, rhizobia

## Abstract

A bioreporter system based on sfYFP, sfCFP, and mScarlet-I in *Mesorhizobium japonicum* and sfYFP and sfCFP in *Rhizobium leguminosarum*, driven by *nifH* consensus promoters was established to monitor occupancy and nitrogen fixation events within legume host nodules. These bioreporter plasmids expand established rhizobium sfGFP system to new hosts and fluorophores.

## ANNOUNCEMENT

A highly competitive but ineffective N_2_-fixing rhizobia strain can outcompete a highly effective but less competitive strain during nodule occupancy, leading to reduced N_2_ fixation (1). This makes competitiveness a key determinant of rhizobial symbiosis (2). We developed a plasmid-based strain labeling system that facilitates evaluation of nodulation competitiveness by quantifying heterologous fluorescent signals from bioreporters in the root nodule, which are expressed by rhizobia during nitrogen fixation. Moreover, fluorescent proteins driven by nitrogenase promoters can serve as bioreporters of nitrogenase activity (1). To expand available tools in this arena, we have extended an established sfGFP-based NifH bioreporter system in *Rhizobium leguminosarum* bv. *viciae 3841-Pisum sativum* indeterminate nodule symbiosis (1) to include additional fluorophores (sfYFP and sfCFP). We newly established this system in the determinate nodule-forming strain *Mesorhizobium japonicum* R7A and the legume *Lotus japonicus* L. cv. Gifu.

**Fig 1:**
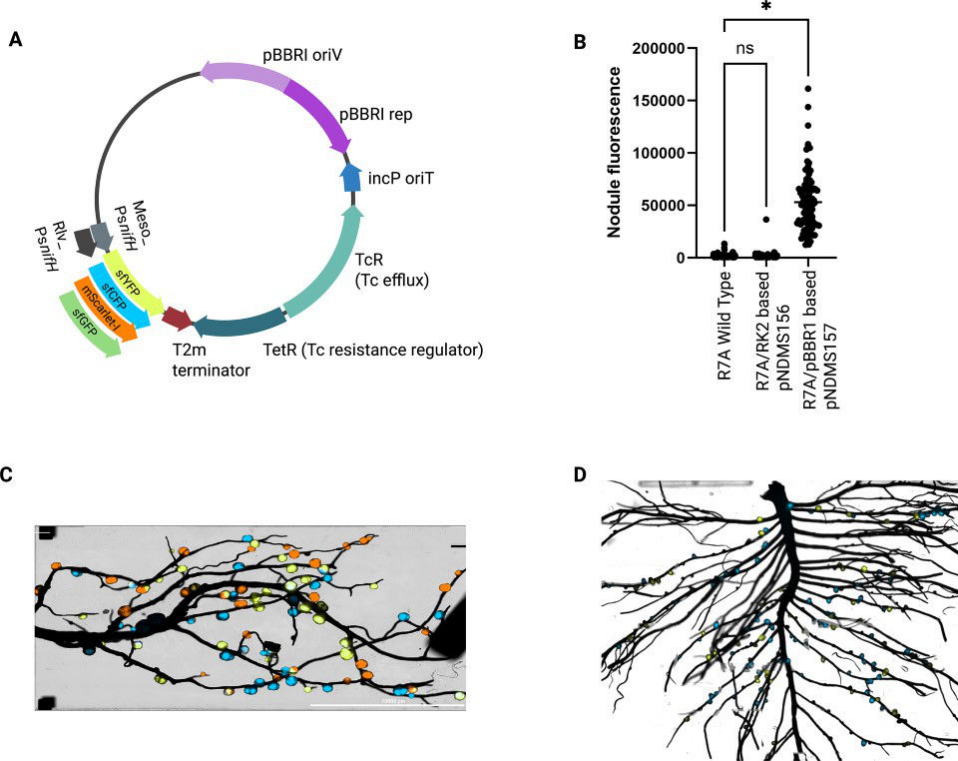
Plasmid map designed using BioRender.com, showing integrated components (Table 1), and graphs of nodule fluorescence from different plasmid backbones expressing sfGFP, and pictures of mixed-inoculated *L. japonicus* and *P. sativum* roots. (**A**) Fluorescent reporter plasmid map showing overall architecture. The construct contains modular, exchangeable promoter (Rlv_*P*s*nifH* or Meso_Ps*nifH*) and fluorescent protein (FP) cassette (sfYFP, sfCFP, mScarlet-I or GFP), enabling flexible customization of gene expression and reporter output. Additional plasmid features include the pBBRI origin of replication (pNDGG004) and selectable antibiotic resistance marker. (**B**) sfGFP nodule fluorescence levels measured using UV transilluminator (Bulldog Bio, Inc.) across different *M. japonicum* strains carrying the bioreporter system with two plasmid backbones: pNDMS156 (pNDGG003) and pNDMS157 (pNDGG004), compared with non-fluorescent R7A. (**C**) *L. japonicus* nodules with mixed-inoculated *M. japonicum* strains expressing sfYFP, sfCFP, and mScarlet-I. (**D**) *P. sativum* nodules with mixed-inoculated *R. leguminosarum* strains expressing sfYFP and sfCFP. Nodules shown in panels **C** and **D** were imaged using the Cytation 5 (BioTek) system according to the methods described in Gautam et al. (5).

Plasmids were constructed by Golden Gate cloning to combine *nifH* promoters with fluorescent proteins and transcriptional terminators from CIDAR (3) within BEVA replicating plasmid backbones (4) (Fig. 1, Table 1). We used a previously developed *Rhizobium* consensus promoter Rlv_Ps*nifH* (pOGG043) (1) and a newly designed synthetic consensus *nifH* promoter for *Mesorhizobium*, Meso_Ps*nifH* (pNDGG076) (Table 1) (5). Plasmids were combined into either RK2 or pBBR1 (Fig. 1A) derived backbones with *par* origins of replication (pNDGG003 and pNDGG004) (4, 6). Golden Gate assembly of these components followed standard procedures. The plasmids were introduced to rhizobia by conjugation (7, 8) and tested for function in root nodules by inoculating *P. sativum* or *L. japonicus* with plasmid-bearing *R. leguminosarum* or *M. japonicum,* respectively, followed by fluorescence microscopy. Stable expression of an analogous sfGFP bioreporter has been established in *Rhizobium* (1). In *Mesorhizobium* where the optimal plasmid backbone was ambiguous, we evaluated RK2 and pBBR1 backbones via Meso_Ps*nifH* sfGFP expression in nodules and observed superior performance of pBBR1 (pNDMS157), as indicated by robust nodule fluorescence (Fig. 1B). Leveraging the pBBR1 plasmid backbone, we generated a series of new plasmids expressing the heterologous fluorophores sfYFP, sfCFP, and mScarlet-I from the Meso_Ps*nifH* promoter (pNDMS237-239) and the Rlv_Ps*nifH* promoter (pNDMS290-292). We found this set of plasmids to be effective in simultaneous assessment of nodulation/nitrogen-fixation events by multiple strains in lotus and pea, respectively (Fig. 1C and D). The usage of these plasmids was robustly explored in an accompanying manuscript (5), where it was found that they functioned effectively to monitor strain identity and nodule fluorescence levels in mixed-inocula experiments with the exception of Rlv_Ps*nifH*-driven mScarlet-I. The tested plasmids that we recommend for community use (pNDMS237, pNDMS238, pNDMS239, pNDMS290, and pNDMS291) were deposited to Addgene.

**TABLE 1:** Fluorescent plasmids and the Golden Gate components

Name	Description	Source	AddGene Plasmid #
C3m	Superfolder green fluorescent protein (sfGFP), Golden Gate component; Addgene CIDAR MoClo Vol.1 Extension (kit #1000,000161); Amp^R^	Richard Murray Lab: CIDAR MoClo Extension Unpublished	120956
C51m	Superfolder yellow fluorescent protein (sfYFP), Golden Gate component; Addgene CIDAR MoClo Vol. 1 Extension (kit #1000000161); Amp^R^	Richard Murray Lab: CIDAR MoClo Extension Unpublished	120975
C91m	Superfolder cyan fluorescent protein (sfCFP), Golden Gate component; Addgene CIDAR MoClo Vol. 1 Extension (kit #1000000161); Amp^R^	Richard Murray Lab: CIDAR MoClo Extension Unpublished	120999
C99m	mScarlet-I, Golden Gate component; Addgene CIDAR MoClo Vol.1 Extension (kit #1000000161); Amp^R^	Richard Murray Lab: CIDAR MoClo Extension Unpublished	121003
pNDGG003	BEVA 2.0 level 1 Golden Gate cloning vector with Bsa1 exchangeable lacZ, RK2 origin of replication, and par stability, Tc^R^	(6)	231316
pNDGG004	BEVA 2.0 level 1 Golden Gate cloning vector with Bsa1 exchangeable lacZ, pBBR1 origin of replication, and par stability, Tc^R^	(6)	231317
pNDGG037	BEVA 2.0 T2m terminator with DF extension CIDAR MoClo GG; Sp^R^	(6)	231337
pOGG043	Synthetic *Rhizobium* P*nifH* Golden Gate component Rlv_Ps*nifH*; Sp^R^	(1)	133123
pNDGG076	Synthetic *Mesorhizobium* P*nifH* PU/AB Golden Gate component Meso_Ps*nifH*; Amp^R^	This work, (5)	NA^*a*^
pNDMS156	Level 1 Golden Gate Cloning with pNDGG003 as backbone with parts: Meso_Ps*nifH*-sfGFP-T2m; Tc^R^	This work	NA
pNDMS157	Level 1 Golden Gate Cloning with pNDGG004 as backbone with parts: Meso_Ps*nifH*-sfGFP-T2m; Tc^R^	This work	NA
pNDMS237	Level 1 Golden Gate Cloning with pNDGG004 as backbone with parts: Meso_Ps*nifH*-sfYFP-T2m; Tc^R^	This work	248095
pNDMS238	Level 1 Golden Gate Cloning with pNDGG004 as backbone with parts: Meso_Ps*nifH*-sfCFP-T2m; Tc^R^	This work	248094
pNDMS239	Level 1 Golden Gate Cloning with pNDGG004 as backbone with parts: Meso_Ps*nifH*-mScarlet-I-T2m; Tc^R^	This work	248096
pNDMS290	Level 1 Golden Gate Cloning with pNDGG004 as backbone with parts: Rlv_Ps*nifH*-sfYFP-T2m; Tc^R^	This work	248097
pNDMS291	Level 1 Golden Gate Cloning with pNDGG004 as backbone with parts: Rlv_Ps*nifH*-sfCFP-T2m; Tc^R^	This work	248098
pNDMS292	Level 1 Golden Gate Cloning with pNDGG004 as backbone with parts: Rlv_Ps*nifH*-mScarlet-I-T2m; Tc^R^	This work	NA

^*a*^ NA indicates not available in AddGene.

Statistical significance of nodule fluorescence levels (Fig. 1B) was assessed using one-way ANOVA followed by Dunnett’s multiple comparisons test in GraphPad Prism (version 10.5.0), with values presented relative to the R7A wild-type control. *P* < 0.05.

## Data Availability

Plasmids pNDMS237, pNDMS238, pNDMS239, pNDMS290, and pNDMS291 have been deposited to Addgene, deposit number 86710 https://www.addgene.org/Barney_Geddes/.
